# Supplementary material for: Identification and evaluation of novel intergenic sites in the vaccinia virus genome for transgene insertion
Source: Mol Ther Oncol. 2026 Jan 19;34(1):201131. doi: 10.1016/j.omton.2026.201131 (PMC12887270; doi:10.1016/j.omton.2026.201131)
Supplement: Document S1. Tables S1 and S2 [file mmc1.pdf]

OMTON, Volume *34*

Supplemental information

Identification and evaluation of novel intergenic sites in the vaccinia virus genome for transgene insertion

Carmen Bueno-Merino, Ana del Canizo, Miquel Conesa, Maria Barcia, Ignacio Sallent, Joan Manils, Sonia-Vanina Forcales, Concepció Soler, and Juan J. Rojas

Table S1. List of intergenic regions between R and L ORFs oriented in opposite sense within VACV WR genome. Following a deep analysis of the VACV WR strain genome, 26 intergenic regions (along with the previously described *I8R-G1L* intergenic region) were identified as candidates based on defined selection criteria. In the left column, gene pairs are listed where the upstream gene is oriented toward the right end (R), and the downstream gene is oriented toward the left end (L). The right column indicates whether the genes overlap or the number of nucleotides (nt) separating them. Three intergenic regions, highlighted in gray, were selected for evaluation as potential insertion sites. These regions were chosen based on the following criteria: 1- no overlap between the flanking R and L genes; 2- short intergenic distance, suggesting minimal risk of disrupting functional elements; 3- high sequence homology of the flanking genes with VACV Copenhagen (Cop), CVA, and MVA strains, enhancing the potential for cross-strain applicability.

Intergenic site	Description
C11R-C10L	152nt
K7R-F1L	overlapping
F17R-E1L	overlapping
E8R-E9L	6nt
E10R-E11L	overlapping
G2R-G4L	overlapping
G6R-G7L	overlapping
L2R-L3L	overlapping
J4R-J5L	72nt
J6R-H1L	overlapping
H2R-H3L	2nt
D1R-D2L	overlapping
D7R-D8L	overlapping
D10R-D11L	0nt
A5R-A6L	overlapping
A8R-A9L	verlapping
A11R-A12L	1nt
A18R-A19L	overlapping
A24R-A25L	4nt
A31R-A32L	overlapping
A37R-A38L	281nt
A40R-A41L	58nt
A43R-A44L	336nt
A46R-A47L	165nt
A52R-A54L	142nt
B16R-B17L	46nt

Table S2. List of oligonucleotides used in the qRT-PCR assay. Random primers were employed for reverse transcription of coding RNAs from genes located upstream of the intergenic regions. In contrast, specific primers targeting the downstream genes were used for the reverse transcription of their coding RNAs to prevent interference from RNA transcripts derived from eGFP expression at the insertion site.

Primer	Sequence 5'3'	Description
qGFP-FW	GACCACTACCAGCAGAACAC	Forward primer to quantify by qPCR eGFP gene expression
qGFP-RV	CCATGTGATCGCGCTTCT	Reverse primer to quantify by qPCR eGFP gene expression
qI8R-FW	AGGATCGCAAGAATTTATTTC	Forward primer to quantify by qPCR *I8R* gene expression
qI8R-RV	TTCTGAATCTATTCGCTGTATAG	Reverse primer to quantify by qPCR *I8R* gene expression
qG1L-FW	ATCATGTCCATGAGAGACGCG	Forward primer to quantify by qPCR *G1L* gene expression
qG1L-RV	TTCTTGACGCAACCAATGATGG	Reverse primer to quantify by qPCR *G1L* gene expression
qD10L-FW	ATTTGCGAGGATGACTCTC	Forward primer to quantify by qPCR *D10R* gene expression
qD10L-RV	AACTATCTCGTCTGTTACATAC	Reverse primer to quantify by qPCR *D10R* gene expression
qD11L-FW	GACGAATGGCTCTTCCTACTATC	Forward primer to quantify by qPCR *D11L* gene expression
qD11L-RV	GCGAGGGTATTAGCTTCTTCTC	Reverse primer to quantify by qPCR *D11L* gene expression
qE8R-FW	GTCTAAGTAGACCGTTAATG	Forward primer to quantify by qPCR *E8R* gene expression
qE8R-RV	AAGTTTGCGTATCTACTACTCC	Reverse primer to quantify by qPCR *E8R* gene expression
qE9L-FW	TTCAGACAACATCTCAGACAG	Forward primer to quantify by qPCR *E9L* gene expression
qE9L-RV	ATGAAATACTCGGCATCGTC	Reverse primer to quantify by qPCR *E9L* gene expression
qH2R-FW	TATAGTAGGCGTACAAGCAG	Forward primer to quantify by qPCR *H2R* gene expression
qH2R-RV	AAGACTGGATTATCAGACG	Reverse primer to quantify by qPCR *H2R* gene expression
qH3L-FW	TTGTCCATTACAAGCTCGG	Forward primer to quantify by qPCR *H3L* gene expression
qH3L-RV	TATTACGTTTCTTCGTCCC	Reverse primer to quantify by qPCR *H3L* gene expression
qGAPDH-FW	ACATCGCTCAGACACCAT	Forward primer to quantify by qPCR GAPDH gene expression
qGAPDH-RV	CAACAATATCCACTTTACCAGAGTT	Reverse primer to quantify by qPCR GAPDH gene expression
rt-G1L	AATCCTTTCTTCTTGAGATCC	Forward primer for the reverse transcription of *G1L* gene
rt-D11L	ATCATGTAGATAGTTACCGTC	Forward primer for the reverse transcription of *D11L* gene
rt-E9L	TAGATTGACTATTTCGGACG	Forward primer for the reverse transcription of *E9L* gene
rt-H3L	TAAGGAACCATAACAGTTTAG	Forward primer for the reverse transcription of *H3L* gene
rt-GAPDH	AGGCATTGCTGATGATCTT	Reverse primer for the reverse transcription of GAPDH gene
