# Supplementary material for: Identification and evaluation of novel intergenic sites in the vaccinia virus genome for transgene insertion
Source: Mol Ther Oncol. 2026 Jan 19;34(1):201131. doi: 10.1016/j.omton.2026.201131 (PMC12887270; doi:10.1016/j.omton.2026.201131)
Supplement: Document S2. Article plus supplemental information [file mmc2.pdf]

Molecular Therapy
Oncology
Brief report

Identification and evaluation of novel intergenic sites in the vaccinia virus genome for transgene insertion

Carmen Bueno-Merino,[1,2] Ana del Canizo,[1,2] Miquel Conesa,[1,2] Maria Barcia,[1,2] Ignacio Sallent,[1,2] Joan Manils,[1,2] Sonia-Vanina Forcales,[1,2] Concepció Soler,[1,2] and Juan J. Rojas[1,2]

[1]Immunology Unit, Department of Pathology and Experimental Therapies, School of Medicine, University of Barcelona – UB, 08907 L'Hospitalet de Llobregat, Spain;
[2]Immunity, Inflammation, and Cancer Group, Oncobell Program, Institut d'Investigació Biomèdica de Bellvitge - IDIBELL, 08908 L'Hospitalet de Llobregat, Spain

Vaccinia virus (VACV) is widely used as a vaccine and oncolytic vector due to its safety profile and its capacity to accommodate exogenous DNA. However, its compact genome offers limited *loci* for transgene insertion without disrupting functional viral sequences. To develop VACV vectors capable of delivering multiple therapeutic genes, additional insertion sites that support stable transgene expression are needed. In this study, we validated the suitability of two new intergenic sites for this purpose. Using *in silico* analysis, we identified candidate sites based on genomic features and the conservation of flanking genes across VACV strains. Using a reporter transgene expression *cassette*, we assessed the potential of these newly identified sites to support transgene expression. Our results demonstrated that the *D10R-D11L* and *E8R-E9L* intergenic sites enable stable and robust transgene expression without diminishing the expression of adjacent viral genes. The modified viruses retained their ability to replicate and kill cancer cells and exhibited similar pathogenicity in mouse models compared to control viruses. These findings identify the *D10R-D11L* and *E8R-E9L* intergenic regions as promising insertion sites for developing VACV-based clinical candidates carrying multiple therapeutic transgenes.

INTRODUCTION

Vaccinia virus (VACV) is a double-stranded DNA virus from the *Poxviridae* family and was historically used as a live virus vaccine to eradicate smallpox.[1] Thanks to its success and strong safety record in this vaccination campaign, as well as the diverse features of various strains, VACV is now widely used in a broad range of therapeutic applications.[2,3] Different VACV strains, such as Western Reserve (WR) or Copenhagen (Cop), are used to develop promising oncolytic viruses due to their rapid lytic cycle, capacity to infect tumor cells of various origins, and ability to elicit antitumor immune responses.[4] Alternatively, VACV strains with restricted replication in mammalian hosts, such as Modified Vaccinia virus Ankara (MVA), are used as safe vectors for delivering antigens from heterologous pathogens or tumors.[3]

A key advantage in all VACV-based therapeutic applications is its large capacity to accommodate foreign DNA.[5] The incorporation and expression of different transgenes enable both antigen delivery for vaccination and enhancement of the antitumor properties of oncolytic vectors. Classically, foreign DNA has been inserted into VACV by replacing nonessential genes *in vitro*, including thymidine kinase (TK), hemagglutinin (HA), ribonucleotide reductase (RR), the VACV growth factor gene, and the *A46R*, *B8R*, and *B14R* genes.[6–10] Although VACV appears to contain a significant amount of genetic material that is dispensable for replication in cell culture, some of these genes may play essential functions *in vivo*. Their deletion could potentially alter the efficacy of the vector,[11–13] particularly in the context of oncolytic therapy. In the MVA strain, major deletion sites have been used for transgene insertion, although genetic instability has been observed in some cases.[14] This approach, however, is unfeasible for oncolytic vectors, as these major deletions are absent. As an alternative, the insertion of foreign DNA into intergenic regions has been explored. Different transgenes have been successfully cloned between the VACV genes *I8R* and *G1L* in both vaccination[15] and oncolytic contexts,[16] demonstrating high transgene stability and minimal impact on viral characteristics. However, the new generation of oncolytic vectors intended for clinical use requires the combination of multiple therapeutic transgenes. Thus, novel, viable intergenic insertion sites are needed for the construction of this new class of therapeutic agents.

In this study, we identify and test novel candidate intergenic sites within the VACV genome for use in both vaccine and oncolytic vector development. We selected three intergenic regions as potential sites for foreign DNA insertion and constructed oncolytic VACV strains with an eGFP expression cassette inserted into these *loci*. We then evaluated their genomic stability, oncolytic properties,

Received 8 July 2025; accepted 13 January 2026;
https://doi.org/10.1016/j.omton.2026.201131.

Correspondence: Juan J. Rojas, Department of Pathology and Experimental Therapies, University of Barcelona, Feixa Llarga, s/n, 08907 Hospitalet de Llobregat, Spain.
E-mail: jrojas@ub.edu

© 2026 The Author(s).
Published by Elsevier Inc. on behalf of The American Society of Gene and Cell Therapy.
This is an open access article under the CC BY license (http://creativecommons.org/licenses/by/4.0/).

and expression levels of the inserted transgene and neighboring genes. Our results show that the regions between the VACV genes *D10R-D11L* and *E8R-E9L* are convenient transgene insertion sites and could be used across a broad spectrum of VACV strains for the generation of multi-transgene clinical candidates.

RESULTS

Identification of intergenic regions within the VACV genome with potential for transgene insertion

The goal of our study was to identify and evaluate new intergenic sites within the VACV genome suitable for the insertion of multiple therapeutic transgenes. First, we performed an *in silico* analysis of various VACV strain genomes to identify intergenic regions with the desired characteristics. Given that the VACV genome is densely packed with coding regions and to minimize disruption of unidentified regulatory elements, we restricted our search to specific intergenic regions. In particular, we focused on regions located between convergently transcribed genes—those where the upstream gene is transcribed rightward (R, according to the VACV Cop strain nomenclature)[17] and the downstream gene leftward (L), with stop codons in close proximity. The intergenic region between the VACV *I8R* and *G1L* genes (Figure 1A) meets these criteria and has previously demonstrated its suitability for stable transgene insertion without detrimental effects on vector characteristics.[15] Our analysis identified 26 such intergenic regions across most VACV genomes, where the upstream open reading frame (ORF) is transcribed rightward and the downstream ORF leftward. Of these, only five included short intergenic sequences of fewer than 40 nucleotides with no gene overlap (Table S1). For this study, we selected three candidate regions based on the conservation of their intergenic sequences and flanking genes across VACV strains commonly used for vector development (Cop, WR, CVA [Chorioallantois Vaccinia virus Ankara], and MVA). The selected intergenic regions *D10R-D11L*, *E8R-E9L*, and *H2R-H3L* are shown in Figure 1A.

Construction of oncolytic VACVs harboring an eGFP expression *cassette* integrated into novel intergenic regions

To assess the suitability of these newly identified intergenic sites, we constructed a set of oncolytic VACVs with an eGFP expression cassette (driven by the strong late VACV promoter P11) inserted into each of the selected regions. A construct with the cassette inserted into the previously validated *I8R-G1L* intergenic site served as a control. The resulting viruses and controls (WR/TK-, WR/TK-/D10R-eGFP-D11L, WR/TK-/E8R-eGFP-E9L, WR/TK-/H2R-eGFP-H3L, and WR/TK-/I8R-eGFP-G1L) are depicted in Figure 1B. As the backbone, we used a VACV WR strain virus with an inactivated TK gene to achieve selective replication in cancer cells,[18] along with an mCherry expression cassette inserted into the TK locus as a fluorescence marker of viral gene expression.

Genetic stability of eGFP expression *cassette* inserted into different VACV intergenic regions

First, we evaluated the genetic stability of the recombinant viruses by confirming the presence of the transgene within the VACV genome through PCR amplification after serial rounds of infection. The results demonstrated that the recombinant viruses with eGFP inserted between *I8R-G1L*, *D10R-D11L*, and *E8R-E9L* remained stable for up to 10 serial passages, with no evidence of deletions or recombination events (Figure 2A). In contrast, insertion of the cassette between *H2R* and *H3L* proved unstable, as bands indicating loss of the transgene were detected after the fifth passage.

Expression levels of eGFP inserted into the identified intergenic regions

Next, we assessed eGFP expression levels using multiple approaches. Quantification of eGFP mRNA by quantitative reverse-transcription PCR (qRT-PCR) revealed increased expression when the cassette was inserted between *D10R-D11L* and *E8R-E9L* compared to the control *I8R-G1L* insertion site (Figure 2B). In contrast, insertion between *H2R* and *H3L* resulted in more than a 7-fold reduction in expression relative to the *I8R-G1L* control. Consistent results were obtained when eGFP fluorescence intensity was measured in infected cells by flow cytometry: similar or increased fluorescence levels were observed for insertions at *D10R-D11L* and *E8R-E9L*, while a significant decrease was detected with the *H2R-H3L* insertion (Figure 2C).

Impact of transgene insertion on viral and oncolytic properties

To further assess the suitability of the newly identified intergenic regions as insertion sites, we evaluated whether transgene insertion affected the functional properties of the recombinant viruses. We first analyzed by qRT-PCR whether foreign DNA insertion influenced the expression of adjacent genes. As shown in Figure 3A, insertion of the *cassette* between *D10R-D11L* and *E8R-E9L* did not reduce the expression of the neighboring genes compared to a virus without foreign DNA inserted in those regions. In contrast, insertion between *H2R* and *H3L* significantly affected the expression of both flanking genes. Interestingly, *cassette* insertion between *I8R* and *G1L* also led to reduced expression of these VACV genes.

We next examined the ability of the recombinant VACVs to replicate in and kill cancer cells. Insertion of the eGFP *cassette* between *D10R-D11L* and *E8R-E9L* did not significantly impair viral replication compared to the unmodified control (Figure 3B). In contrast, insertions at *H2R-H3L* and *I8R-G1L* negatively impacted this feature. Despite these effects, all recombinant viruses retained their ability to kill tumor cells effectively (Figure 3C).

Finally, we monitored mouse body weight after systemic administration of the recombinant viruses to determine whether genomic modifications altered *in vivo* virulence. All viruses induced comparable weight-loss profiles. However, mice treated with the virus carrying the insertion between *I8R* and *G1L* showed slightly reduced weight loss by day 4, suggesting mild attenuation (Figure 3D). The virus with the transgene insertion between *H2R* and *H3L* was excluded from this experiment as its impairment had been clearly demonstrated in previous experiments.

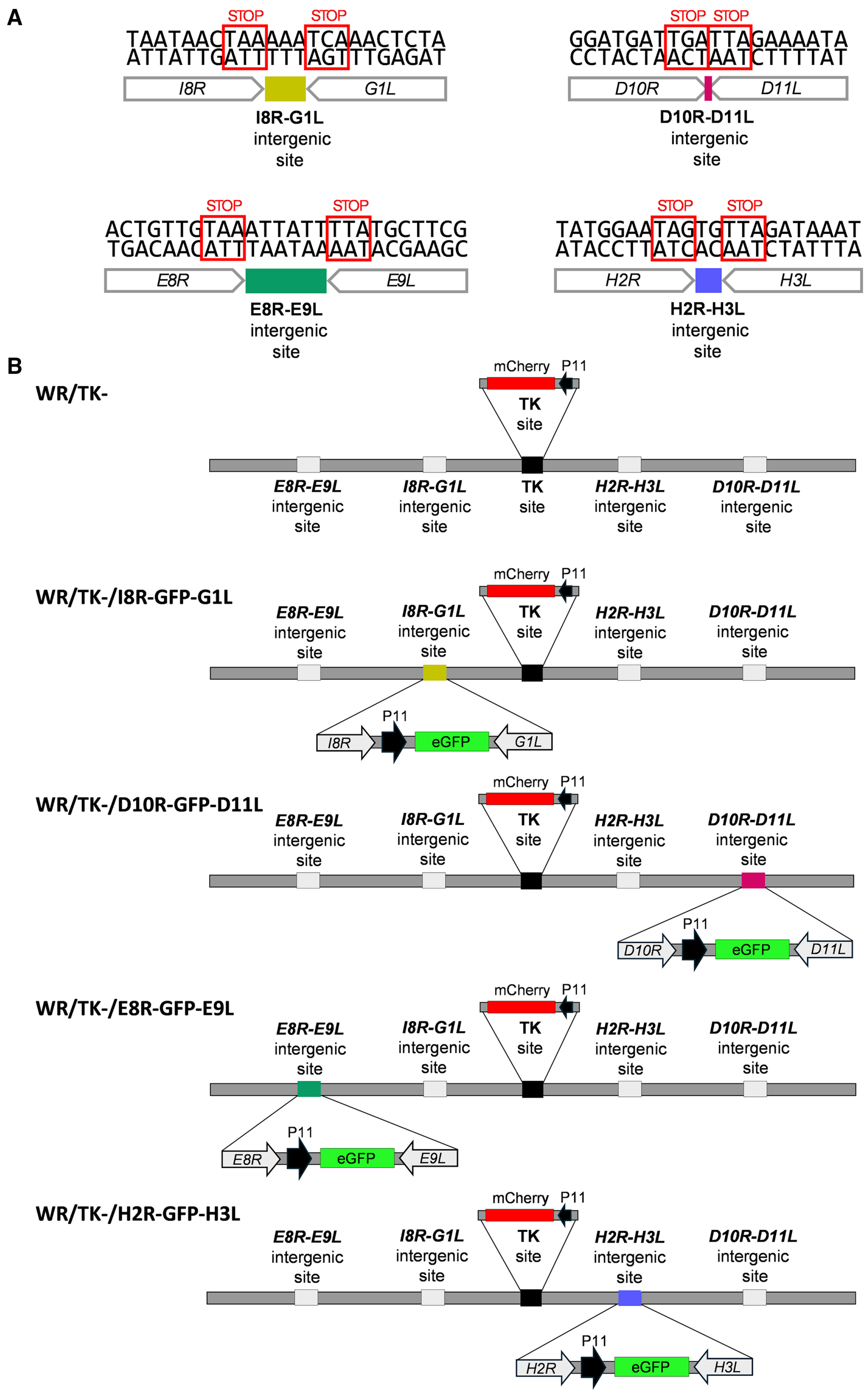

Figure 1. Generation of oncolytic VACV vectors expressing eGFP from newly identified intergenic regions

(A) Schematic representation of the four intergenic regions within the VACV WR strain genome used in this study for transgene insertion: *I8R-G1L*, *D10R-D11L*, *E8R-E9L*, and *H2R-H3L*. All intergenic regions are short, non-overlapping intergenic sequences located between ORFs transcribed in opposite orientations. (B) Diagram of the different recombinant VACV constructs generated in this study, showing insertion of the P11-eGFP expression *cassette* into the indicated intergenic regions. WR/TK- was used as the backbone for these modifications and served as a control in viral characterization assays.

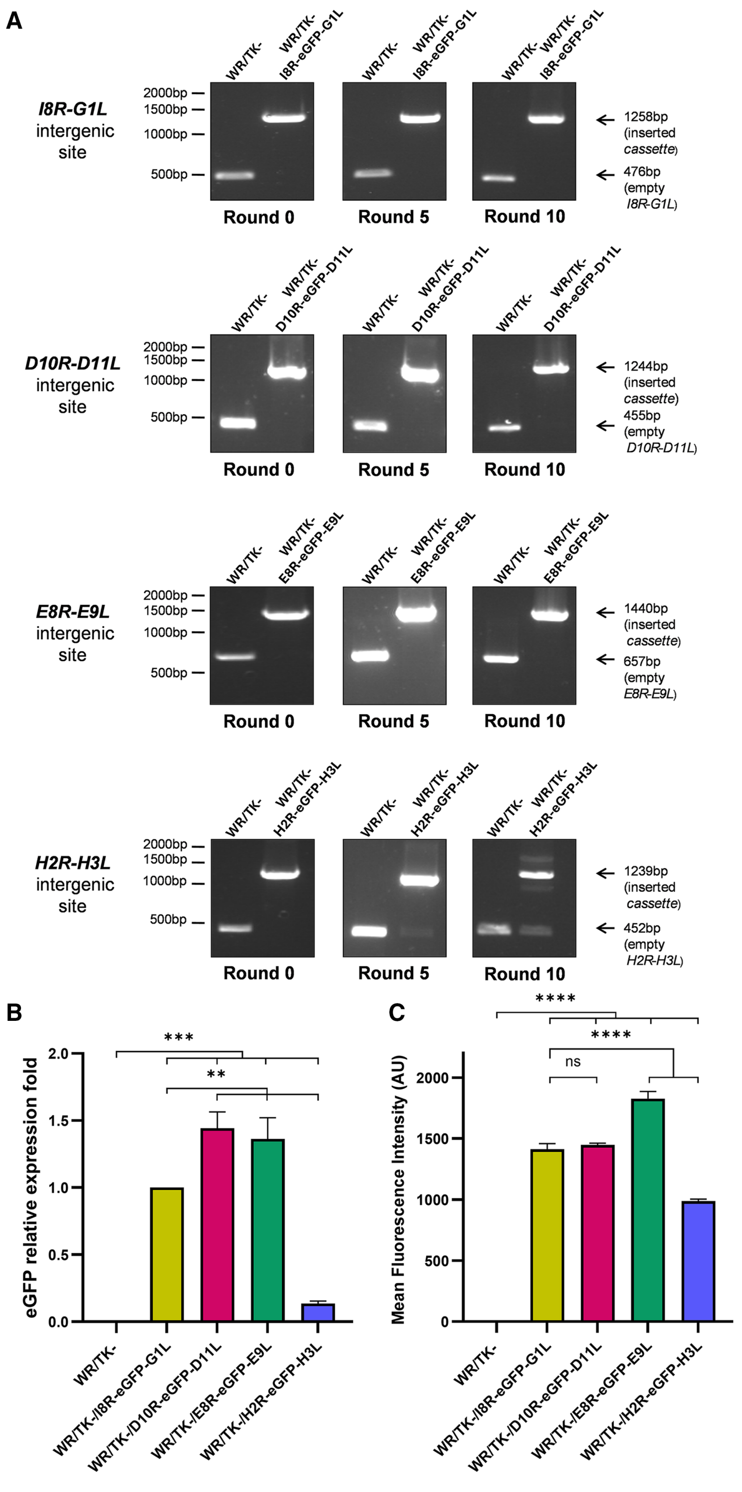

Figure 2. Transgene stability and quantification of transgene expression

(A) PCR analysis of transgene insert stability after 10 serial passages. Expected amplicon sizes for each recombinant virus are indicated. (B) Relative eGFP mRNA expression levels. HeLa cells were infected at an MOI of 20, and total RNA was extracted 6 h post-infection. eGFP mRNA levels were quantified by qRT-PCR and normalized to GAPDH using the $2^{-\Delta\Delta Cq}$ method. (C) Quantification of eGFP fluorescence by flow cytometry 24 h post-infection. For both (B) and (C), data are presented as mean ± SD from three independent biological replicates. ns, not significant; **, $p < 0.01$; ***, $p < 0.001$; ****, $p < 0.0001$.

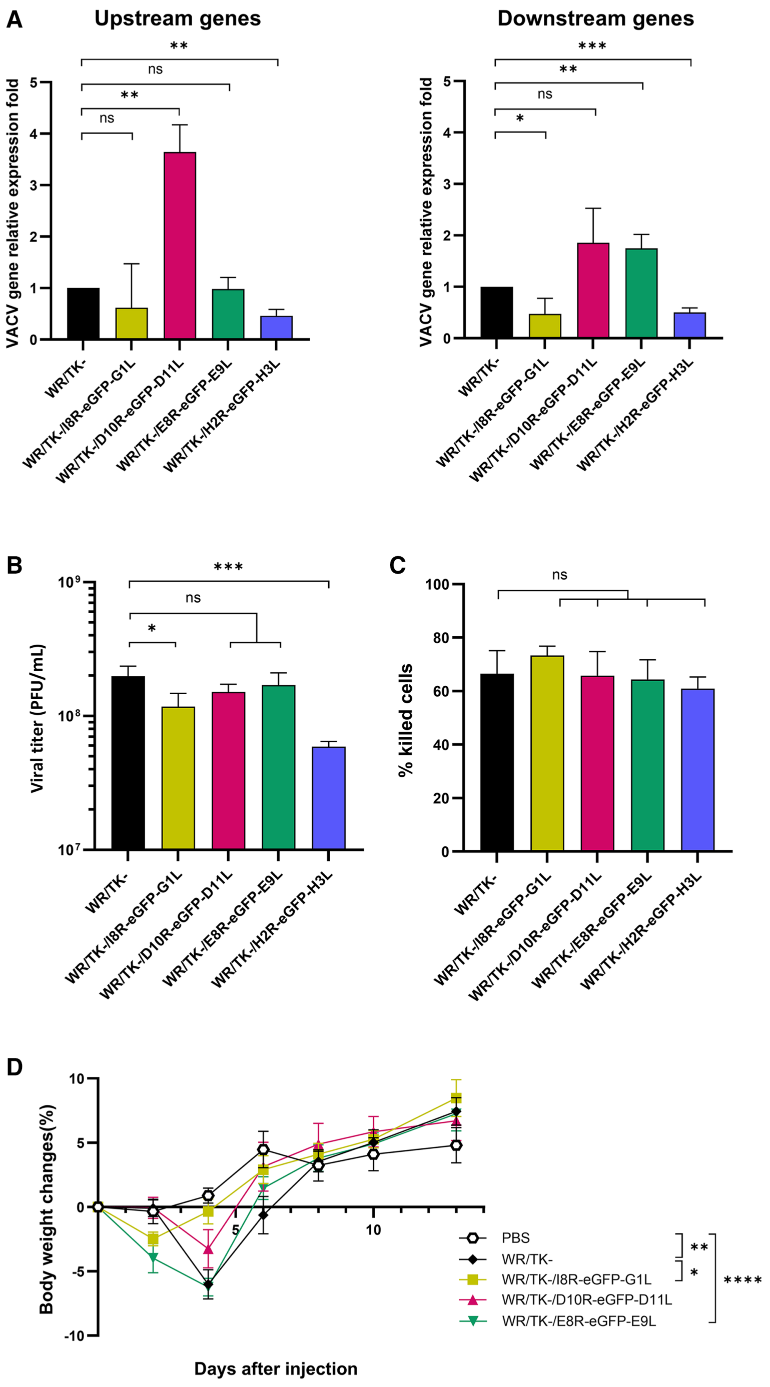

Figure 3. Functional characterization of recombinant VACVs

(A) Relative expression of genes flanking the insertion sites. HeLa cells were infected with the indicated viruses at an MOI of 20, and total RNA was extracted 6 h post-infection. mRNA expression levels of upstream (*I8R*, *D10R*, *E8R*, *H2R*) and downstream (*G1L*, *D11L*, *E9L*, *H3L*) genes for each intergenic region were quantified by qRT-PCR and normalized to GAPDH using the $2^{-\Delta\Delta Cq}$ method. Data are plotted as fold change versus WR/TK- + SD from three independent biological replicates. (B) Comparative viral replication. HeLa cells were infected at an MOI of 5 and viral titers were determined by plaque assays 48 h post-infection. (C) Cytotoxicity in HeLa cells. Cells were infected at an MOI of 1 with the indicated viruses and the percentage of dead cells was quantified 72 h post-infection. For both (B) and (C), data represent the mean ± SD from three independent biological replicates. (D) Body weight changes following intravenous administration. BALB/c mice were injected intravenously with 1×10^8 PFU of indicated viruses, and body weight was monitored every 2 days until full recovery. PBS was used as a control. Data represent the mean ± SEM for five individual mice, and significance is shown for day 4 post-injection. ns, not significant; *, $p < 0.05$; **, $p < 0.01$; ***, $p < 0.001$; ****, $p < 0.0001$.

DISCUSSION

In this study, we describe and evaluate novel intergenic regions within the VACV genome as potential insertion sites for therapeutic transgenes. We specifically selected intergenic regions flanked by convergently oriented genes—where the upstream gene is transcribed rightward and the downstream gene leftward—to minimize the potential disruption of regulatory elements. This strategy has proven successful with the intergenic region between the *I8R* and *G1L* VACV genes.[15] The intergenic region between the VACV genes *I4L* and *I5L* (both transcribed leftward) has also been previously evaluated in the context of the non-replicative strain MVA, but increased immunogenicity was detected, suggesting altered expression of neighboring genes.[19]

To assess the suitability of our selected intergenic regions for transgene insertion, we used eGFP as a reporter, allowing straightforward quantification of transgene expression. Our results indicate that the *E8R-E9L* and *D10R-D11L* intergenic regions are ideal for this purpose as foreign DNA insertion between these genes did not critically impair the expression of adjacent genes or affect viral replication and virulence. Moreover, these genomic modifications were stable, and the inserted transgene was expressed at high levels. In contrast, the third intergenic site tested in this study, *H2R-H3L*, showed limited potential for transgene insertion, as impaired viral replication and reduced expression levels for both the transgene and flanking genes were observed. Notably, our results also show that insertion of foreign DNA between *I8R-G1L*, which has been previously used for transgene insertion,[15,16] negatively impacts viral replication and *in vivo* virulence. Although eGFP represents a relatively small transgene, we anticipate that moderate increases in insert size—such as those typical of single therapeutic genes—are unlikely to substantially affect transgene expression, genetic stability, or virus replication.

Our results also suggest that the suitability of a VACV intergenic region for transgene insertion is not linked to the function of the flanking genes, but rather to how the insertion affects their expression. All VACV genes flanking the tested insertion sites are essential for processes such as entry, replication, transcription, or virion assembly.[20–27] However, introduction of foreign DNA into the intergenic region only significantly and negatively impacted the expression levels of VACV genes *G1L*, *H2R*, and *H3L*. Interestingly, DNA insertion between *D10R-D11L* and *E8R-E9L* increased the expression levels of *D10R*, *D11L*, and *E9L*, although no effect on viral replication or virulence was observed. The size of the flanking genes and their timing of expression during the VACV life cycle do not appear to influence the impact of transgene insertion either. Thus, our experimental findings are highly relevant for researchers aiming to optimize transgene placement in the design of clinical-grade VACV vectors.

This study was conducted in the context of oncolytic vectors, as this is the therapeutic approach for VACV that stands to benefit the most from multiple transgene expression. Since both robust replication and elicitation of immune responses are key for the antitumor efficacy of oncolytic VACVs,[11] the cloning of transgenes into these agents should not compromise these features by deleting VACV genes with potential or unexpected effects *in vivo*. Therefore, the availability of multiple intergenic insertion sites that do not compromise replication or virulence is of high value. The construction of clinical candidates that combine the expression of transgenes to improve antitumor immunity,[16,28] overcome barriers imposed by the tumor microenvironment,[29] or activate prodrugs[30] could result in effective responses in tumors that are currently resistant to immunotherapies. Moreover, other clinical applications, such as the construction of vaccination vectors, may also benefit from these novel insertion sites. Although these *loci* were selected based on their conservation across VACV strains, empirical validation in strains other than WR could provide additional support for the transferability of these findings.

In summary, the intergenic regions between VACV genes *E8R-E9L* and *D10R-D11L* represent novel, advantageous sites for transgene insertion and can be used for the development of multitransgene VACV vectors for diverse therapeutic applications.

MATERIALS AND METHODS

In silico genome analysis

Benchling software was used to identify intergenic regions between two adjacent ORFs coding in opposite directions, with the upstream ORF oriented rightward and the downstream ORF oriented leftward. The published sequence of VACV strain WR (GenBank: AY243312.1) was used for this analysis. The identified target regions were compared with the published sequences of VACV strains Cop (GenBank: M35027.1), CVA (GenBank: AM501482.1), and MVA (GenBank: AY603355.1).

Cell lines and viruses

HeLa and BSC-40 cell lines were purchased from the American Type Culture Collection (ATCC) and maintained in the recommended culture media supplemented with 5%–10% fetal bovine serum and 1% penicillin-streptomycin at 37°C with 5% CO_2.

All recombinant VACVs used or generated in this work are based on the VACV strain WR. Construction of WR/TK-, which includes a truncated viral TK gene and expresses mCherry under the VACV-specific late promoter (P11), was previously described.[4] For the construction of WR/TK-/I8R-eGFP-G1L, WR/TK-/D10R-eGFP-D11L, WR/TK-/E8R-eGFP-E9L, and WR/TK-/H2R-eGFP-H3L, a P11-eGFP expression *cassette* was inserted into the specified intergenic sites by homologous recombination. The targeted regions were located between the viral genes *I8R* and *G1L*, *D10R* and *D11L*, *E8R* and *E9L*, and *H2R* and *H3L*, respectively. The recombinant viruses were isolated using a selection process based on eGFP expression and purified by ultracentrifugation through sucrose cushions, as previously described.[18] Viral titers were determined by plaque assay in BSC-40 cells.

Genomic stability of recombinant viruses

HeLa cells were initially infected with the indicated viruses at an MOI of 0.05 and, 2 days after infection, samples were harvested and subjected to three freeze-thaw cycles in preparation for the subsequent infection round. The infection process was serially repeated up to 10 times, and viral DNA was isolated at rounds 0, 5, and 10 using the QIAamp DNA Micro Kit (Qiagen) for PCR analysis of the modified regions.

Flow cytometry

HeLa cells were infected with the indicated viruses at an MOI of 5, and samples were collected and fixed with 4% paraformaldehyde 24 h after infection. eGFP fluorescence intensity was analyzed using a FACSCanto flow cytometer (BD Biosciences) and FlowJo software.

mRNA expression analysis

HeLa cells were infected with the indicated viruses at an MOI of 20. Samples were harvested 6 h after infection, and total RNA was purified using the NucleoSpin RNA-Protein Extraction Kit (Macherey-Nagel). Three independent replicates were performed for each condition. Reverse transcription for cDNA synthesis was carried out using the High-Capacity cDNA Reverse Transcription Kit (Applied Biosystems), following the manufacturer's instructions. Random or gene-specific primers (5 μM) were used with 1 μg of RNA as a template for each reaction. For the qRT-PCR analysis, 1 μL of the reverse transcription product was amplified using SYBR Select Master Mix (Applied Biosystems) in the presence of specific primers (5 μM) under the following conditions: an initial denaturation step at 95°C for 10 min, followed by 40 cycles of 95°C for 15 s, 55°C for 35 s, and 72°C for 40 s, with a final melting curve from 60°C to 95°C at 0.075°C per cycle. The analysis was performed on a QuantStudio 7 Pro quantitative real-time PCR system (Thermo Fisher Scientific), and relative gene expression levels were normalized to GAPDH using the 2-ΔΔCq method. Primers used are listed in Table S2.

Virus growth and cytotoxicity assays

To test viral replication, HeLa cells were infected with the indicated viruses at an MOI of 5. Two hours post-infection, the viral-containing medium was removed and fresh medium was added to the cells. After 48 h, samples were collected, subjected to three freeze-thaw cycles, and viral titters were determined by plaque assay in BSC-40 cells.

For cytotoxicity assays, HeLa cells were infected with the indicated viruses at an MOI of 1, and 3 days after infection, a non-radioactive cell proliferation assay was used to quantify surviving cells and the percentage of cell death.

Mouse models

All animal experiments were approved by the local ethics committee of the University of Barcelona. Six-week-old female BALB/c mice were obtained from Charles River Laboratories and housed in isolated cage units with *ad libitum* access to food and water. Mice were randomized and the indicated viruses were administered intravenously at a dose of 1×10^8 plaque-forming unit (PFU) per mouse. Each group consisted of 10 animals. Body weight was monitored every 2 days until day 13 post-virus injection, when full recovery of weight was observed.

Statistical analyses

The results presented in Figures 2B, 2C, 3B, and 3C were analyzed using a one-way ANOVA followed by Tukey's multiple-comparison test. A standard Student's t test was applied to the results shown in Figure 3A, using an independent WR/TK- condition as the specific control for each gene. To study mouse weight loss, a two-way ANOVA followed by a Bonferroni post hoc test were used. Statistical significance was defined as $p < 0.05$ for all analyses.

DATA AND CODE AVAILABILITY

The data presented in this study are available upon request from the corresponding author.

ACKNOWLEDGMENTS

This work was supported by the AECC Scientific Foundation (FC_AECC LAB-AE223473ROJA) and by grant PID2022-138519OB-I00 (JJR), funded by MICIU/AEI/10.13039/501100011033 (Spanish Ministry of Sciences) and by FEDER, EU. It was also supported by the Catalan Government (2021-SGR-00275, JJR) and by the Carlos III Health Institute (ISCIII) through the Spanish Network of Advanced Therapies (TERAV) (project RD21/0017/0012). JJR was funded through the Ramon y Cajal program (RYC2018-025425-I), financed by MCIN/AEI/10.13039/501100011033 and by "ESF Investing in your future." We thank the CERCA Programme/Generalitat de Catalunya for institutional support. The graphical abstract was generated using BioRender.

AUTHOR CONTRIBUTIONS

Conceptualization, C.B.-M. and J.J.R.; methodology, J.M., S.V.F., C.S., and J.J.R.; investigation, C.B.-M., A.D.C., M.C., M.B., and I.S.; writing – original draft, C.B.-M.; writing – review & editing, J.J.R. and I.S.; supervision, J.M., S.V.F., C.S., and J.J.R.; funding acquisition, J.J.R.

DECLARATION OF INTERESTS

The authors declare no conflict of interest.

DECLARATION OF GENERATIVE AI AND AI-ASSISTED TECHNOLOGIES IN THE WRITING PROCESS

During the preparation of this work, the authors used ChatGPT Plus in order to improve readability and language. After using this tool, the authors reviewed and edited the content as needed and take full responsibility for the content of the publication.

SUPPLEMENTAL INFORMATION

Supplemental information can be found online at https://doi.org/10.1016/j.omton.2026.201131.

REFERENCES

1. Fenner, F. (1989). Risks and benefits of vaccinia vaccine use in the worldwide smallpox eradication campaign. Res. Virol. *140*, 465–491. https://doi.org/10.1016/s0923-2516(89)80126-8.

2. Guo, Z.S., Lu, B., Guo, Z., Giehl, E., Feist, M., Dai, E., Liu, W., Storkus, W.J., He, Y., Liu, Z., and Bartlett, D.L. (2019). Vaccinia virus-mediated cancer immunotherapy: cancer vaccines and oncolytics. J. Immunother. Cancer *7*, 6. https://doi.org/10.1186/s40425-018-0495-7.

3. Volz, A., and Sutter, G. (2017). Modified Vaccinia Virus Ankara: History, Value in Basic Research, and Current Perspectives for Vaccine Development. Adv. Virus Res. *97*, 187–243. https://doi.org/10.1016/bs.aivir.2016.07.001.

4. Riederer, S., Fux, R., Lehmann, M.H., Volz, A., Sutter, G., and Rojas, J.J. (2021). Activation of interferon regulatory factor 3 by replication-competent vaccinia viruses improves antitumor efficacy mediated by T cell responses. Mol. Ther. Oncolytics *22*, 399–409. https://doi.org/10.1016/j.omto.2021.06.001.

5. Smith, G.L., and Moss, B. (1983). Infectious poxvirus vectors have capacity for at least 25 000 base pairs of foreign DNA. Gene *25*, 21–28. https://doi.org/10.1016/0378-1119(83)90163-4.

6. Norton, A., Peplinski, G.R., and Tsung, K. (1996). Expression of secreted platelet-derived growth factor-B by recombinant nonreplicating and noncytopathic vaccina virus. Ann. Surg. *224*, 555–562. https://doi.org/10.1097/00000658-199610000-00013.

7. Garcia-Arriaza, J., Gomez, C.E., Sorzano, C.O., and Esteban, M. (2014). Deletion of the vaccinia virus N2L gene encoding an inhibitor of IRF3 improves the immunogenicity of modified vaccinia virus Ankara expressing HIV-1 antigens. J. Virol. *88*, 3392–3410. https://doi.org/10.1128/JVI.02723-13.

8. Orubu, T., Alharbi, N.K., Lambe, T., Gilbert, S.C., and Cottingham, M.G. (2012). Expression and cellular immunogenicity of a transgenic antigen driven by endogenous poxviral early promoters at their authentic loci in MVA. PLoS One *7*, e40167. https://doi.org/10.1371/journal.pone.0040167.

9. Boulton, S., Poutou, J., Gill, R., Alluqmani, N., He, X., Singaravelu, R., Crupi, M.J.F., Petryk, J., Austin, B., Angka, L., et al. (2023). A T cell-targeted multi-antigen vaccine generates robust cellular and humoral immunity against SARS-CoV-2 infection. Mol. Ther. Methods Clin. Dev. *31*, 101110. https://doi.org/10.1016/j.omtm.2023.101110.

10. Whelan, J.T., Singaravelu, R., Wang, F., Pelin, A., Tamming, L.A., Pugliese, G., Martin, N.T., Crupi, M.J.F., Petryk, J., Austin, B., et al. (2022). CRISPR-mediated rapid arming of poxvirus vectors enables facile generation of the novel immunotherapeutic STINGPOX. Front. Immunol. *13*, 1050250. https://doi.org/10.3389/fimmu.2022.1050250.

11. Rojas, J.J., Van Hoecke, L., Conesa, M., Bueno-Merino, C., Del Canizo, A., Riederer, S., Barcia, M., Brosinski, K., Lehmann, M.H., Volz, A., et al. (2024). A new MVA ancestor-derived oncolytic vaccinia virus induces immunogenic tumor cell death and robust antitumor immune responses. Mol. Ther. *32*, 2406–2422. https://doi.org/10.1016/j.ymthe.2024.05.014.

12. Sroller, V., Ludvíková, V., Maresová, L., Hainz, P., and Německová, S. (2001). Effect of IFN-gamma receptor gene deletion on vaccinia virus virulence. Arch. Virol. *146*, 239–249. https://doi.org/10.1007/s007050170172.

13. Chen, R.A.J., Jacobs, N., and Smith, G.L. (2006). Vaccinia virus strain Western Reserve protein B14 is an intracellular virulence factor. J. Gen. Virol. *87*, 1451–1458. https://doi.org/10.1099/vir.0.81736-0.

14. Neckermann, P., Mohr, M., Billmeier, M., Karlas, A., Boilesen, D.R., Thirion, C., Holst, P.J., Jordan, I., Sandig, V., Asbach, B., and Wagner, R. (2024). Transgene expression knock-down in recombinant Modified Vaccinia virus Ankara vectors improves genetic stability and sustained transgene maintenance across multiple passages. Front. Immunol. *15*, 1338492. https://doi.org/10.3389/fimmu.2024.1338492.

15. Wyatt, L.S., Earl, P.L., Xiao, W., Americo, J.L., Cotter, C.A., Vogt, J., and Moss, B. (2009). Elucidating and minimizing the loss by recombinant vaccinia virus of human immunodeficiency virus gene expression resulting from spontaneous mutations and positive selection. J. Virol. *83*, 7176–7184. https://doi.org/10.1128/JVI.00687-09.

16. Van Hoecke, L., Riederer, S., Saelens, X., Sutter, G., and Rojas, J.J. (2020). Recombinant viruses delivering the necroptosis mediator MLKL induce a potent antitumor immunity in mice. OncoImmunology *9*, 1802968. https://doi.org/10.1080/2162402X.2020.1802968.

17. Kaynarcalidan, O., Moreno Mascaraque, S., and Drexler, I. (2021). Vaccinia Virus: From Crude Smallpox Vaccines to Elaborate Viral Vector Vaccine Design. Biomedicines *9*, 1780. https://doi.org/10.3390/biomedicines9121780.

18. Riederer, S., Del Canizo, A., Navas, J., Peter, M.G., Link, E.K., Sutter, G., and Rojas, J.J. (2023). Improving poxvirus-mediated antitumor immune responses by deleting viral cGAMP-specific nuclease. Cancer Gene Ther. *30*, 1029–1039. https://doi.org/10.1038/s41417-023-00610-5.

19. Manuel, E.R., Wang, Z., Li, Z., La Rosa, C., Zhou, W., and Diamond, D.J. (2010). Intergenic region 3 of modified vaccinia ankara is a functional site for insert gene expression and allows for potent antigen-specific immune responses. Virology *403*, 155–162. https://doi.org/10.1016/j.virol.2010.04.015.

20. Bayliss, C.D., and Smith, G.L. (1996). Vaccinia virion protein I8R has both DNA and RNA helicase activities: implications for vaccinia virus transcription. J. Virol. *70*, 794–800. https://doi.org/10.1128/JVI.70.2.794-800.1996.

21. Hedengren-Olcott, M., Byrd, C.M., Watson, J., and Hruby, D.E. (2004). The vaccinia virus G1L putative metalloproteinase is essential for viral replication in vivo. J. Virol. *78*, 9947–9953. https://doi.org/10.1128/JVI.78.18.9947-9953.2004.

22. Senkevich, T.G., and Moss, B. (2005). Vaccinia virus H2 protein is an essential component of a complex involved in virus entry and cell-cell fusion. J. Virol. *79*, 4744–4754. https://doi.org/10.1128/JVI.79.8.4744-4754.2005.

23. da Fonseca, F.G., Wolffe, E.J., Weisberg, A., and Moss, B. (2000). Effects of deletion or stringent repression of the H3L envelope gene on vaccinia virus replication. J. Virol. *74*, 7518–7528. https://doi.org/10.1128/jvi.74.16.7518-7528.2000.

24. Cao, S., Molina, J.A., Cantu, F., Hernandez, C., and Yang, Z. (2022). A Poxvirus Decapping Enzyme Colocalizes with Mitochondria To Regulate RNA Metabolism and Translation and Promote Viral Replication. mBio *13*, e0030022. https://doi.org/10.1128/mbio.00300-22.

25. Koonin, E.V., and Senkevich, T.G. (1992). Vaccinia virus encodes four putative DNA and/or RNA helicases distantly related to each other. J. Gen. Virol. *73*, 989–993. https://doi.org/10.1099/0022-1317-73-4-989.

26. Kato, S.E.M., Condit, R.C., and Moussatché, N. (2007). The vaccinia virus E8R gene product is required for formation of transcriptionally active virions. Virology *367*, 398–412. https://doi.org/10.1016/j.virol.2007.05.002.

27. Czarnecki, M.W., and Traktman, P. (2017). The vaccinia virus DNA polymerase and its processivity factor. Virus Res. *234*, 193–206. https://doi.org/10.1016/j.virusres.2017.01.027.

28. Azar, F., Deforges, J., Demeusoit, C., Kleinpeter, P., Remy, C., Silvestre, N., Foloppe, J., Fend, L., Spring-Giusti, C., Quéméneur, E., and Marchand, J.B. (2024). TG6050, an oncolytic vaccinia virus encoding interleukin-12 and anti-CTLA-4 antibody, favors tumor regression via profound immune remodeling of the tumor microenvironment. J. Immunother. Cancer *12*, e009302. https://doi.org/10.1136/jitc-2024-009302.

29. Wang, S., Li, Y., Xu, C., Dong, J., and Wei, J. (2024). An oncolytic vaccinia virus encoding hyaluronidase reshapes the extracellular matrix to enhance cancer chemotherapy and immunotherapy. J. Immunother. Cancer *12*, e008431. https://doi.org/10.1136/jitc-2023-008431.

30. Ding, Y., Fan, J., Deng, L., Huang, B., and Zhou, B. (2020). Antitumor efficacy of cytosine deaminase-armed vaccinia virus plus 5-fluorocytosine in colorectal cancers. Cancer Cell Int. *20*, 243. https://doi.org/10.1186/s12935-020-01340-6.

OMTON, Volume *34*

Supplemental information

Identification and evaluation of novel intergenic sites in the vaccinia virus genome for transgene insertion

Carmen Bueno-Merino, Ana del Canizo, Miquel Conesa, Maria Barcia, Ignacio Sallent, Joan Manils, Sonia-Vanina Forcales, Concepció Soler, and Juan J. Rojas

Table S1. List of intergenic regions between R and L ORFs oriented in opposite sense within VACV WR genome. Following a deep analysis of the VACV WR strain genome, 26 intergenic regions (along with the previously described *I8R-G1L* intergenic region) were identified as candidates based on defined selection criteria. In the left column, gene pairs are listed where the upstream gene is oriented toward the right end (R), and the downstream gene is oriented toward the left end (L). The right column indicates whether the genes overlap or the number of nucleotides (nt) separating them. Three intergenic regions, highlighted in gray, were selected for evaluation as potential insertion sites. These regions were chosen based on the following criteria: 1- no overlap between the flanking R and L genes; 2- short intergenic distance, suggesting minimal risk of disrupting functional elements; 3- high sequence homology of the flanking genes with VACV Copenhagen (Cop), CVA, and MVA strains, enhancing the potential for cross-strain applicability.

Intergenic site	Description
C11R-C10L	152nt
K7R-F1L	overlapping
F17R-E1L	overlapping
E8R-E9L	6nt
E10R-E11L	overlapping
G2R-G4L	overlapping
G6R-G7L	overlapping
L2R-L3L	overlapping
J4R-J5L	72nt
J6R-H1L	overlapping
H2R-H3L	2nt
D1R-D2L	overlapping
D7R-D8L	overlapping
D10R-D11L	0nt
A5R-A6L	overlapping
A8R-A9L	verlapping
A11R-A12L	1nt
A18R-A19L	overlapping
A24R-A25L	4nt
A31R-A32L	overlapping
A37R-A38L	281nt
A40R-A41L	58nt
A43R-A44L	336nt
A46R-A47L	165nt
A52R-A54L	142nt
B16R-B17L	46nt

Table S2. List of oligonucleotides used in the qRT-PCR assay. Random primers were employed for reverse transcription of coding RNAs from genes located upstream of the intergenic regions. In contrast, specific primers targeting the downstream genes were used for the reverse transcription of their coding RNAs to prevent interference from RNA transcripts derived from eGFP expression at the insertion site.

Primer	Sequence 5'3'	Description
qGFP-FW	GACCACTACCAGCAGAACAC	Forward primer to quantify by qPCR eGFP gene expression
qGFP-RV	CCATGTGATCGCGCTTCT	Reverse primer to quantify by qPCR eGFP gene expression
qI8R-FW	AGGATCGCAAGAATTTATTTC	Forward primer to quantify by qPCR *I8R* gene expression
qI8R-RV	TTCTGAATCTATTCGCTGTATAG	Reverse primer to quantify by qPCR *I8R* gene expression
qG1L-FW	ATCATGTCCATGAGAGACGCG	Forward primer to quantify by qPCR *G1L* gene expression
qG1L-RV	TTCTTGACGCAACCAATGATGG	Reverse primer to quantify by qPCR *G1L* gene expression
qD10L-FW	ATTTGCGAGGATGACTCTC	Forward primer to quantify by qPCR *D10R* gene expression
qD10L-RV	AACTATCTCGTCTGTTACATAC	Reverse primer to quantify by qPCR *D10R* gene expression
qD11L-FW	GACGAATGGCTCTTCCTACTATC	Forward primer to quantify by qPCR *D11L* gene expression
qD11L-RV	GCGAGGGTATTAGCTTCTTCTC	Reverse primer to quantify by qPCR *D11L* gene expression
qE8R-FW	GTCTAAGTAGACCGTTAATG	Forward primer to quantify by qPCR *E8R* gene expression
qE8R-RV	AAGTTTGCGTATCTACTACTCC	Reverse primer to quantify by qPCR *E8R* gene expression
qE9L-FW	TTCAGACAACATCTCAGACAG	Forward primer to quantify by qPCR *E9L* gene expression
qE9L-RV	ATGAAATACTCGGCATCGTC	Reverse primer to quantify by qPCR *E9L* gene expression
qH2R-FW	TATAGTAGGCGTACAAGCAG	Forward primer to quantify by qPCR *H2R* gene expression
qH2R-RV	AAGACTGGATTATCAGACG	Reverse primer to quantify by qPCR *H2R* gene expression
qH3L-FW	TTGTCCATTACAAGCTCGG	Forward primer to quantify by qPCR *H3L* gene expression
qH3L-RV	TATTACGTTTCTTCGTCCC	Reverse primer to quantify by qPCR *H3L* gene expression
qGAPDH-FW	ACATCGCTCAGACACCAT	Forward primer to quantify by qPCR GAPDH gene expression
qGAPDH-RV	CAACAATATCCACTTTACCAGAGTT	Reverse primer to quantify by qPCR GAPDH gene expression
rt-G1L	AATCCTTTCTTCTTGAGATCC	Forward primer for the reverse transcription of *G1L* gene
rt-D11L	ATCATGTAGATAGTTACCGTC	Forward primer for the reverse transcription of *D11L* gene
rt-E9L	TAGATTGACTATTTCGGACG	Forward primer for the reverse transcription of *E9L* gene
rt-H3L	TAAGGAACCATAACAGTTTAG	Forward primer for the reverse transcription of *H3L* gene
rt-GAPDH	AGGCATTGCTGATGATCTT	Reverse primer for the reverse transcription of GAPDH gene
